# Presentation of Kaposi Sarcoma With Extensive Skin Lesions and Bone Involvement After Ten Years of Virally Suppressed HIV Infection and Restored CD4 Cell Counts

**DOI:** 10.1155/crdi/6183442

**Published:** 2026-11-15

**Authors:** Kwong Tack Quach, Eliane Leyten, Frank Jeurissen, Rachel Roach, Koen Van Der Bogt, Nico Aarts, Anh Nguyen, Leroy Lard

**Affiliations:** ^1^ Department of Dermatology The Hague Medical Centre The Hague Netherlands; ^2^ Department of Internal Medicine/ Infectious Diseases / Oncology The Hague Medical Centre The Hague Netherlands; ^3^ Department of Surgery The Hague Medical Centre The Hague Netherlands; ^4^ Department of Radiology The Hague Medical Centre The Hague Netherlands; ^5^ Department of Pathology The Hague Medical Centre The Hague Netherlands; ^6^ Department of Rheumatology The Hague Medical Centre The Hague Netherlands

**Keywords:** bone lesions, HIV, Kaposi sarcoma, skin lesions

## Abstract

**Background:**

Kaposi sarcoma (KS) is an HHV‐8‐associated angioproliferative tumor that can occur in persons living with HIV despite effective antiretroviral therapy. Although cutaneous KS after immune restoration has been described, extensive osseous involvement after prolonged viral suppression remains uncommon.

**Case Presentation:**

A 51‐year‐old man with HIV‐1 infection and sustained viral suppression for more than 10 years presented with severe pain in the right foot, progressive swelling of the legs, and skin discoloration. Initial diagnostic considerations included gout, stasis dermatitis, and osteomyelitis. Persistent symptoms despite treatment prompted further investigation. MRI demonstrated multiple pathological bone lesions in both feet. Skin and bone biopsies subsequently confirmed HHV‐8–positive KS involving the skin and bone. At diagnosis, the patient had a CD4 cell count of 750 cells/μL and a CD4/CD8 ratio of 1.42, with an undetectable HIV viral load. Treatment with pegylated liposomal doxorubicin resulted in a substantial clinical and metabolic response, but the patient experienced two subsequent recurrences. Symptomatic localized osseous progression of KS in the left foot was treated with radiotherapy, resulting in significant pain relief, while pegylated liposomal doxorubicin was continued with a favorable clinical and metabolic response.

**Conclusion:**

This case highlights that extensive cutaneous and osseous KS can develop and recur despite prolonged viral suppression and restoration of conventional markers of immune function. KS should therefore remain in the differential diagnosis of unexplained cutaneous lesions, edema, and bone lesions in persons living with HIV, even when CD4 cell counts are preserved and HIV viral load remains undetectable. Long‐term clinical surveillance and individualized multimodal treatment may be required.

## 1. Background

Kaposi sarcoma (KS) is an angioproliferative tumor induced by human herpesvirus‐8 (HHV‐8) [1]. Human immunodeficiency virus (HIV)/AIDS–associated KS is the most common tumor in persons living with HIV (PLHIV) [1]. These patients typically have low CD4 cell counts and a high viral load [2]. The incidence of HIV‐associated KS has decreased significantly since the introduction of highly active antiretroviral therapy (ART) [1]. According to current guidelines, PLHIV directly start ART at the time of diagnosis, independent of their CD4 cell count [3]. In the majority of cases, KS is diagnosed at the time of HIV diagnosis or shortly after the start of ART as a result of immune reconstitution syndrome unmasking previously unnoticed KS. Nonetheless, even in virally suppressed patients with immune restoration, cutaneous KS, although rare, may still develop [2]. In contrast, less is known about the development of KS bone lesions in well‐controlled HIV patients [4]. We present an unusual case of KS of the skin and bone after 10 years of HIV treatment with undetectable viral loads and restored CD4 cell counts.

## 2. Case Presentation

A 51‐year‐old Ethiopian man was diagnosed with HIV‐1 infection in December 2013 after admission for viral meningitis. His medical history consisted of allergic eosinophilic asthma for which he was treated with inhaled ciclesonide, beclometasone/formoterol, and salbutamol. ART was initiated at that time and initially consisted of atazanavir/ritonavir and zidovudine/lamivudine (Combivir). The patient had been regularly followed in our HIV outpatient clinic since diagnosis, with HIV RNA monitored approximately three times per year and consistently remaining undetectable. He was highly adherent to ART.

In 2021, he was diagnosed with crystal‐proven gout of the first metatarsophalangeal joint. Joint aspiration demonstrated intracellular monosodium urate crystals, and serum uric acid was elevated at 0.53 mmol/L (530 μmol/L). He was treated with colchicine followed by allopurinol, with subsequent resolution of his symptoms. He remained free of gout symptoms until November 2022, when he presented with recurrent severe pain in the right foot. Furthermore, he also noted progressive swelling and discoloration of the legs since a couple of months.

During physical examination, lymphedema, pitting edema, and erythematous plaques with postinflammatory hyperpigmentation were observed on the lower legs (Figure 1), and the patient experienced pain at the first digit of the right foot. The initial hypothesis was a gout attack despite adequate uric acid levels, and the edema and discoloration were found to be more consistent with stasis dermatitis. Treatment was initiated with colchicine, three short courses of prednisone (30 mg once daily for five days each), and an increased allopurinol dosage for the gout. There was no history of chronic systemic glucocorticoid use. Compression therapy was started for the stasis dermatitis. This, however, did not improve his symptoms, and the patient developed severe localized pain at the nail bed of the first metatarsophalangeal joint in December 2022. Further examination with an X‐ray raised suspicion of osteomyelitis of the interphalangeal joint, after which empiric treatment with oral high‐dose flucloxacillin was started for 6 weeks, with also no effect.

**FIGURE 1:**
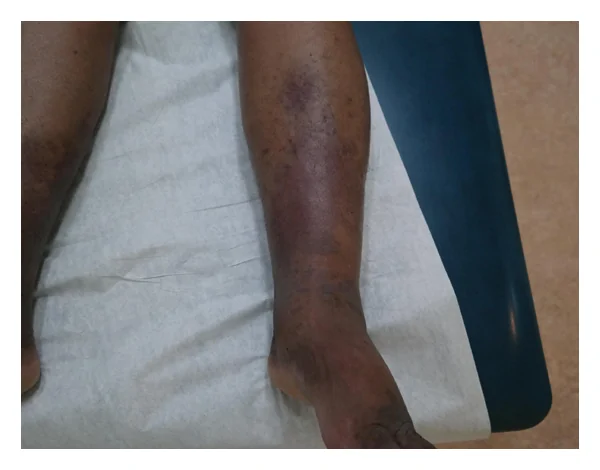
Photograph of the lower extremities at clinical presentation, demonstrating bilateral lymphedema with marked pitting edema, extensive erythematous plaques, and areas of postinflammatory hyperpigmentation.

KS was initially considered to be less likely as he had been successfully treated for HIV since the beginning of diagnosis and was not known to have KS previously. His viral load had been undetectable (< 20 copies/mL) since the start of combination ART (cART), and his immunological parameters at the time of KS diagnosis were as follows: CD4 cell count 750 cells/μL, CD8 cell count 520 cells/μL, and CD4/CD8 ratio 1.42 (nadir CD4 cell count, 220 cells/μL in 2013). Because the symptoms persisted despite adequate gout treatment and compression therapy, further diagnostic evaluation was warranted. Therefore, MRI of both feet was performed, which showed one pathological bone lesion in the first digit of the right foot and two pathological bone lesions in the first and third digits of the left foot and bone marrow edema without signs of arthritis, making an acute gout flare less likely (Figure 2). Subsequently, a skin biopsy was performed in March 2023, which showed an atypical endothelial proliferation with positive immunohistochemical staining for ERG (C‐terminal clone), CD34, and smooth muscle actin (SMA). The initial skin biopsy performed at Haaglanden Medical Center (HMC) showed negative HHV‐8 staining. Upon reanalysis at Leiden University Medical Center (LUMC), HHV‐8 staining was positive in the lesional cells, confirming the diagnosis of KS. In April 2023, a bone biopsy was obtained from the distal phalanx of the right first toe, which showed an atypical spindle cell proliferation with positive immunohistochemical staining for ERG (C‐terminal clone), SMA, CD31, and CD34 and negative HHV‐8 staining in the lesional cells. Because of concerns regarding the reliability of HHV‐8 staining in the decalcified bone specimen, the bone biopsy was reanalyzed at LUMC. Both HMC and LUMC use the Dako Omnis staining platform (Agilent). At LUMC, immunohistochemical stains, including HHV‐8, ERG (N‐terminal clone), SMA, CD31, and CD34, were repeated because of the potentially reduced reliability of staining in decalcified bone specimens. The repeated HHV‐8 staining was positive in the lesional cells, confirming the diagnosis of KS (Figure 3). Plasma testing for HHV‐8 yielded negative results in May 2023. Unfortunately, no prior HHV‐8 testing was available. To evaluate the extent, an FDG‐PET/CT was performed in May 2023, which showed skin lesions in both legs and several bone lesions in the feet, left fibula, and left calcaneus (Figure 4). According to the AIDS Clinical Trials Group (ACTG) staging system, the patient was classified as having T1I0S0 disease. The T1 classification was based on the presence of tumor‐associated lymphedema. The osseous lesions were not considered visceral disease and therefore did not determine the *T* category. The I0 classification was supported by a CD4 cell count of 750 cells/μL at the time of KS diagnosis. The S0 classification reflected the absence of KS‐related systemic illness or other criteria for systemic disease at the time of staging.

**FIGURE 2:**
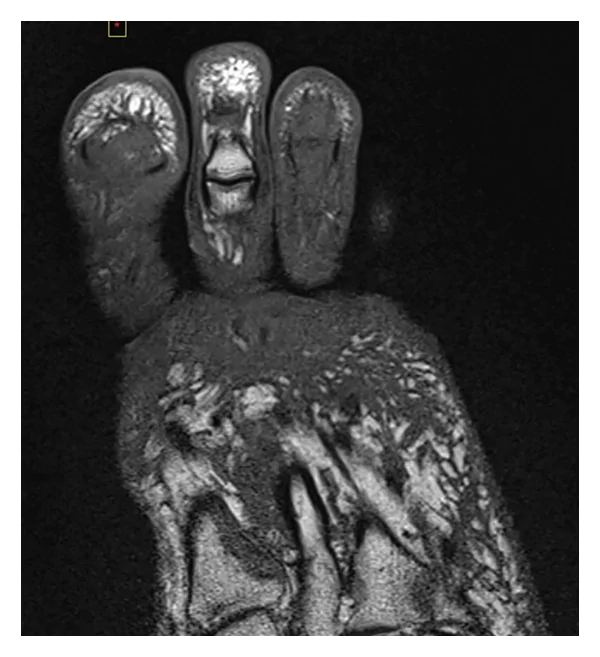
Coronal T1‐weighted magnetic resonance image shows a low signal intensity mass within the distal phalanx with extension into the soft tissue.

**FIGURE 3:**
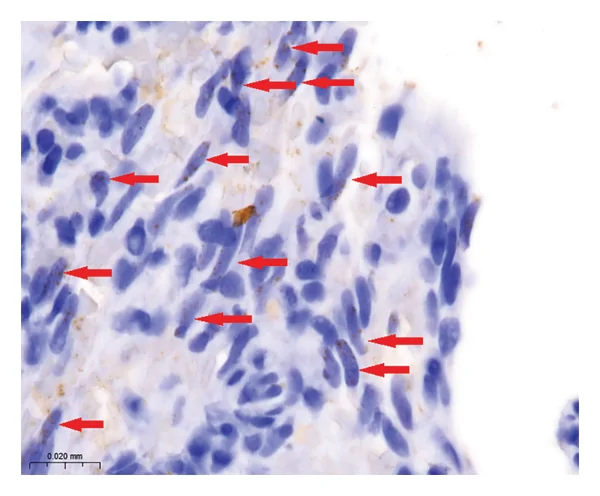
Immunohistochemical staining for human herpesvirus 8 (HHV‐8) in the bone biopsy specimen (first digit of the right foot), demonstrating nuclear positivity in a subset of cells, with positive staining areas highlighted by red arrows.

**FIGURE 4:**
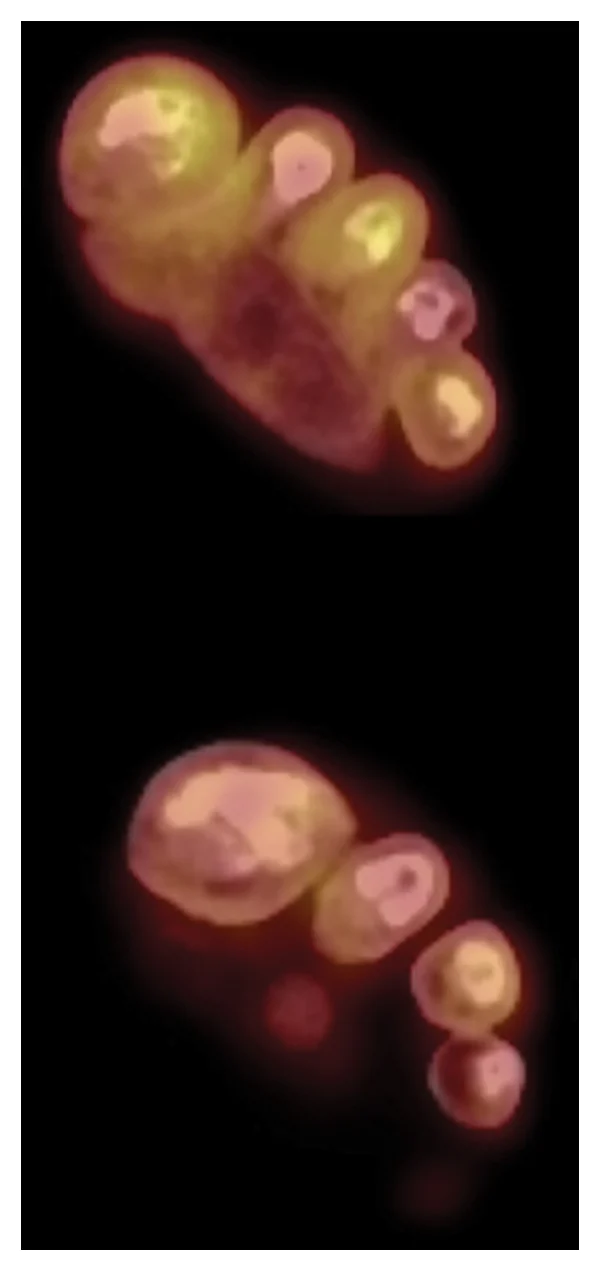
FDG‐PET/CT before (upper panel; May 2023) and after (lower panel; October 2023) seven cycles of pegylated liposomal doxorubicin (PLD; Caelyx) shows a significant reduction in the skin and bone lesions.

Because at that time there was discussion regarding the effect of specific cART classes on HHV‐8 replication and KS progression, we modified his antiretroviral regimen in May 2023 by switching from a non‐nucleoside reverse transcription inhibitor–based regimen to a protease inhibitor–based regimen. Specifically, Delstrigo was discontinued and replaced with darunavir 600 mg in combination with ritonavir 100 mg twice daily, while the backbone of emtricitabine and tenofovir (nucleoside reverse transcriptase inhibitor) was continued. Although current NCCN guidelines do not recommend specific ART class modifications for KS management [3] and recent data suggest that protease inhibitors may have limited impact on KS outcomes [2], this decision was based on several earlier reports suggesting that protease inhibitors may inhibit HHV‐8 replication and reduce KS progression [5–7], in the hope of improving the clinical outcome.

Furthermore, treatment with pegylated liposomal doxorubicin (PLD; Caelyx) was started in June 2023 at a dose of 20 mg/m^2^ every three weeks. Based on a body surface area of 2.11 m^2^, this corresponded to approximately 40 mg administered per cycle in practice. The edema and pain improved significantly, and the FDG‐PET/CT performed in October 2023 after seven cycles of PLD showed a significant reduction in all cutaneous and osseous lesions (Figure 4).

Unfortunately, the patient experienced a recurrence of symptoms and osseous KS lesions in July 2024 despite continued treatment with darunavir/ritonavir and sustained viral suppression, for which PLD was restarted at 20 mg/m^2^ every three weeks, with 40 mg administered per cycle. Treatment was discontinued in December 2024 after eight cycles because of a good clinical response, with remission again demonstrated on FDG‐PET/CT.

Sadly, the patient experienced a second recurrence in June 2025. PLD was restarted because of increasing pain in the left foot, with FDG‐PET/CT demonstrating progression of both cutaneous and osseous lesions involving the feet, lower legs, upper legs, and possibly the right elbow. After three cycles of PLD, the patient experienced worsening pain in the left foot. Follow‐up FDG‐PET/CT showed progression of the osteolytic lesions corresponding to the symptomatic area, while the remaining known lesions were stable or responding to treatment. Because of the localized symptomatic progression in a functionally important area, irradiation of the left foot was performed with five fractions of 4 Gy, aiming to provide pain relief and local tumor control. In KS, radiotherapy is an established and well‐tolerated treatment option for localized disease, particularly in anatomically challenging or functionally important areas [8]. The patient experienced a clear clinical response, with a significant reduction in pain after radiotherapy. Subsequently, systemic treatment with PLD was continued, with the dose reduced to 30 mg per cycle during the final three cycles in November and December 2025 because of oral mucositis. After a total of eight cycles in this treatment period, the patient showed a favorable clinical and metabolic response of all known KS lesions on FDG‐PET/CT performed in January 2026. A clinical timeline summarizing the disease course and treatment is presented in Figure 5.

**FIGURE 5:**
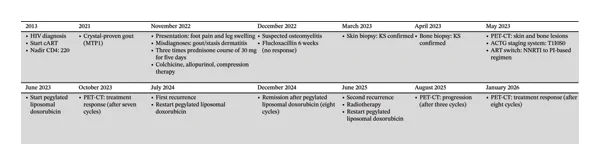
Clinical timeline of the disease course and treatment.

## 3. Discussion

We present an unusual case of cutaneous and osseous KS developing after 10 years of continuous HIV treatment with sustained viral suppression and restored CD4 cell counts. Although the development of osseous KS in patients with well‐controlled HIV infection has previously been described, this case is notable for the prolonged duration of viral suppression, preserved CD4/CD8 ratio, and the relapsing course requiring prolonged systemic and local treatment.

In our HIV clinic, we analyzed 1027 HIV patients in care and found that 33 (3.2%) had a history of KS (unpublished data). In 10 patients (30.3% of all KS patients), KS was found at the time of HIV diagnosis, and in nine patients (27.3% of all KS patients), the KS was diagnosed within six months after the start of cART, which can be considered as unmasking previously unnoticed KS due to an immune reconstitution syndrome. In 12 patients (36.4% of all KS patients), KS diagnosis was more than six months after HIV diagnosis (range: 6 months–14 years). However, all these patients were not on cART and had a detectable viral load at the time of KS diagnosis. Only in two patients (0.2% of all HIV patients), KS developed after years of continuous cART with undetectable viral loads. One is the patient described in this case report, and the other patient (after 14 years of successful HIV treatment) developed KS only after his CD4 cell count had decreased to 150 cells/mm^3^ due to receiving chemotherapy for anal carcinoma, representing a fundamentally different pathophysiological scenario. Therefore, the present case represents the only true occurrence of KS developing during sustained viral suppression and immune restoration in our cohort.

These findings are in accordance with large European HIV cohorts (109.461 HIV patients) showing a KS incidence of 0.8% more than 1 month after starting cART [9]. The incidence of KS was highest in the first 180 days after starting cART (9.5 per 1000 person‐years). Afterward, the incidence declines rapidly 5–8 years after starting cART. In line with our observation, most patients diagnosed with KS more than 1 year after the start of cART had a detectable viral load. Although these large cohorts show that KS is rare in well‐treated HIV patients, it is important to appreciate that the incidence of KS still remains much higher than in the general population.

The development of KS after prolonged viral suppression is therefore uncommon but not previously unrecognized [10]. Several case series and reports have described cutaneous KS in patients with well‐controlled HIV infection, including recurrent episodes of limited cutaneous disease [9, 11–14]. Bone involvement by KS has historically been more commonly reported in advanced cases with high viral loads and severe immunosuppression. However, several reports and case series have documented KS bone lesions occurring even in patients on ART with immune restoration [12, 15, 16].

Caponetti et al. [14] performed an extensive literature review of KS involving the musculoskeletal system, identifying a total of 66 cases, of which 28 patients (42.4%) had AIDS‐associated KS. Their findings revealed that, while bone involvement frequently accompanied cutaneous disease, isolated bone KS was exceedingly rare. The lesions typically involved the peripheral skeleton in African and classic KS cases and the axial and maxillofacial skeleton in AIDS‐associated KS. Patients often presented with pain, and imaging showed osteolytic lesions in most cases. It is important to note that the majority of these patients were diagnosed in the era before the widespread introduction of cART. Only five patients (17.9%) were receiving cART at the time of diagnosis, with poor compliance and low CD4 cell counts (< 100 cells/mm^3^), thus hampering comparability with the present case.

Papanastasopoulos et al. [15] reported on a large cohort of HIV‐infected patients in the modern ART era and found a prevalence of 1.1% of KS involving the skeletal system out of a cohort of 1489 HIV‐infected patients with biopsy‐proven KS. All patients had cutaneous KS at the time of diagnosis, and involvement of the skeletal system occurred only many years later (median: 3.3 years; maximum: 9.2 years), often presenting as lytic lesions with cortical involvement. Interestingly, the majority of patients were asymptomatic at the time when skeletal involvement occurred, which may be attributable to adherence to cART with sustained viral suppression and immune restoration. Most patients had sustained viral suppression and high CD4 counts at the time when skeletal involvement occurred. This pattern is consistent with our case and raises the possibility that bone KS in virally suppressed patients may represent a distinct clinical entity.

Similarly, Verdecia et al. [16] describe a case of a well‐controlled HIV patient who developed a recurrence of KS after 4 years with bone involvement of the right foot despite excellent adherence to cART with viral suppression and immune restoration. Palich et al. [12] also report on four patients with bone KS (18%) despite treatment with cART with viral suppression and immune restoration.

These studies highlight the gradually concerning issue of KS development in well‐controlled HIV patients. Our case adds to this literature primarily through its clinical context and disease course. The patient developed extensive cutaneous and osseous KS after 10 years of sustained viral suppression, considerably longer than the period described in some previous reports, while maintaining a preserved CD4/CD8 ratio. Furthermore, the disease followed a relapsing course despite continuous HIV treatment and required repeated systemic treatment with PLD, as well as local radiotherapy for symptomatic osseous progression. Of note, the patient’s antiretroviral regimen was switched from a non‐nucleoside reverse transcriptase inhibitor–based regimen to darunavir/ritonavir in May 2023, with the aim of potentially improving control of HHV‐8 and reducing KS progression based on earlier reports [5–7]. However, darunavir/ritonavir was continued thereafter, and the patient maintained sustained viral suppression while subsequently developing recurrent osseous KS. This clinical course argues against a meaningful effect of the protease inhibitor–based regimen on KS control in our patient and is consistent with more recent evidence suggesting a limited impact of protease inhibitors on KS outcomes [2, 3]. This prolonged and multimodal disease course illustrates that adequate viral suppression and restoration of conventional markers of immune function do not necessarily eliminate the risk of KS recurrence or progression. Importantly, CD4 cell count and viral load primarily reflect the degree of HIV control and immune restoration, whereas the extent and stage of KS remain important determinants of disease prognosis. Recent data also suggest that higher HHV‐8 viral load may be associated with worse survival in HIV‐associated KS [17].

A possible explanation for KS occurring despite effective ART is HIV‐associated immune senescence. Although long‐term ART effectively suppresses HIV replication and allows substantial recovery of CD4 cell counts, it may not completely reverse the persistent immune activation and immune dysfunction associated with chronic HIV infection. There is increasing evidence that chronic viral infections, such as HIV and cytomegalovirus (CMV), induce senescence of the immune system through persistent immune activation [10]. Markers of immune senescence include a decreased CD4/CD8 ratio, upregulation of immune checkpoint molecules, and an increased frequency of senescent T cells, leading to immune dysregulation. In PLHIV, normalization of the CD4/CD8 ratio is rarely observed, even after early initiation of cART with CD4 cell count recovery. Our patient, however, has had normal CD4/CD8 ratios since 2014 after initiation of ART, which would argue against classical immune senescence. Alternative mechanisms, such as impaired HHV‐8–specific T‐cell response independent of CD4 count, may therefore be more relevant, as recent data suggest that HHV‐8–specific cellular immunity may remain deficient despite immune restoration [2].

There are indications that localized HHV‐8 reactivation may also play a role as patients with both HIV‐related and classic KS have been shown to exhibit a decreased HHV‐8–specific T‐cell response, leading to uncontrolled proliferation of HHV‐8–transformed cells and the subsequent development of KS, regardless of CD4 cell count [18]. This hypothesis is compatible with our findings of positive HHV‐8 staining in both the skin and bone biopsies. Furthermore, age‐related and HIV‐associated inflammaging has been suggested to contribute to impaired immune control of HHV‐8 and subsequent development of KS [10]. The discordance in our case between tissue HHV‐8 positivity and plasma negativity may be explained by the predominantly localized nature of the disease, as circulating HHV‐8 DNA is not consistently detectable in all patients with KS, presumably due to inhibitory effects of ART on circulating HHV‐8 virus [19].

The pattern of repeated responses to PLD followed by relapse after treatment discontinuation may reflect insufficient immune control of HHV‐8 despite immune restoration, rather than primary PLD treatment failure. This observation is in line with recent literature suggesting that KS in virally suppressed patients may follow a relapsing course and pose a therapeutic challenge, potentially reflecting the natural history of disease in this specific population [2].

Our case report underlines the importance of considering KS in PLHIV presenting with skin and bone lesions, even in the setting of effective ART, sustained viral suppression, and restored CD4 cell counts. Early diagnosis is particularly important, as limited disease may allow for local treatment and improve outcomes. This case also illustrates that clinically significant KS may develop and follow a challenging, relapsing course despite sustained viral suppression and restored CD4 cell counts, highlighting an unmet clinical need in PLHIV. Therefore, long‐term clinical surveillance remains warranted, including regular physical examination and ongoing HIV follow‐up. Further research is needed to better characterize the factors associated with KS development and disease course despite effective ART and to identify patients who may benefit from closer monitoring.

In our case, follow‐up was individualized according to symptoms and disease activity. Routine oncology and HIV outpatient visits were scheduled every 3 months, while additional evaluations could be performed earlier in case of increasing pain, progression of cutaneous lesions, edema, or functional decline. Decisions regarding reinitiation of therapy were based, among other factors, on clinical progression. FDG‐PET/CT imaging was obtained before initiation or reinitiation of systemic therapy and after treatment completion for response assessment. Routine PET/CT surveillance was not performed. Current clinical practice does not recommend routine FDG‐PET/CT follow‐up in HIV‐associated KS, and imaging is generally reserved for suspected progressive, visceral, or atypical disease manifestations. However, PET/CT may be helpful in selected cases, particularly in the evaluation of osseous, nodal, muscular, or visceral involvement. Given the marked heterogeneity in clinical presentation and disease course of HIV‐associated KS, no standard follow‐up schedule exists, and follow‐up strategies should be tailored to the individual patient.

In conclusion, the current case represents an emerging clinical challenge requiring further research into its occurrence, surveillance, and new therapeutic strategies beyond conventional ART optimization and chemotherapy.

## Funding

No funding was received for this manuscript.

## Consent

The authors obtained written consent from the patient for their photographs and medical information to be published in print and online, with the understanding that this information may be publicly available. Patient consent forms were not provided to the journal but are retained by the authors.

## Conflicts of Interest

The authors declare no conflicts of interest.

## Data Availability

The data that support the findings of this study are available upon request from the corresponding author. The data are not publicly available due to privacy or ethical restrictions.
